# Poor Skeletal Robustness on Lower Extremities and Weak Lean Mass Development on Upper Arm and Calf: Normal Weight Obesity in Middle-School-Aged Children (9 to 12)

**DOI:** 10.3389/fped.2018.00371

**Published:** 2018-12-06

**Authors:** Martin Musálek, Jana Pařízková, Elena Godina, Elvira Bondareva, Jakub Kokštejn, Jan Jírovec, Šárka Vokounová

**Affiliations:** ^1^Faculty of Physical Education and Sport, Charles University, Prague, Czechia; ^2^Obesity Management Centre, Institute of Endocrinology, Prague, Czechia; ^3^Anuchin Research Institute and Museum of Anthropology, Lomonosov Moscow State University, Moscow, Russia

**Keywords:** normal weight obesity, children, adipose tissue, skeletal robustness, lean mass, body mass index

## Abstract

**Background:** Normal weight obesity in children has been associated with excessive body fat, lower bone density and decreased total lean mass. However, no studies have been done into whether normal weight obese children differ in skeletal robustness or lean mass development on the extremities from normal weight non-obese, overweight, and obese peers although these are important indicators of healthy development of children.

**Methods:** Body height, body weight, BMI, four skinfolds, and two limb circumferences were assessed. We calculated total body fat using Slaughter's equations, the Frame index for skeletal robustness and muscle area for the upper arm and calf using Rolland-Cachera equations. Using national references of BMI and measured skinfolds, three subgroups of participants (9–12 years) consisting of 210 middle-school-aged children (M-age = 11.01 ± 1.05)−110 girls and 100 boys—were selected: (A) overweight obese (OWOB) (*n* = 72); (B) normal weight obese (NWO) (*n* = 69); and, (C) normal weight non-obese (NWNO) (*n* = 69). All values, were converted to Z-scores to take account of participant's sex and age.

**Results:** NWO children had significantly poorer skeletal robustness on lower extremities and poorer muscle area on the upper arm and calf compared to NWNO counterparts with significantly higher evidence in boys–skeletal robustness NWO boys: Z-score = −0.85; NWO girls: Z-score = −0.43; lean mass on the calf: NWO boys Z-score = −1.34; NWO girls: Z-score = −0.85. The highest skeletal robustness—but not muscle area on the calf—was detected in OWOB children.

**Conclusions:** Further research should focus on whether this poor skeletal and lean mass development: (1) is a consequence of insufficient physical activity regimes; (2) affects physical fitness of NWO children and could contribute to a higher prevalence of health problems in them. We have highlighted the importance of the development of a simple identification of NWO children to be used by pediatricians.

## Introduction

Normal weight obesity (1–4) is a state in which an excessive amount of total body fat and a decreased lean mass is accompanied by average/normal BMI values.

Previous research has documented that normal weight obese (NWO) adults can have serious functional, metabolic, and cardiovascular problems (3–7). Romero-Corral et al. (8) revealed that the incidence of the metabolic syndrome in NWO subjects was four times higher than in so-called normosthenic population (normal weight, normal BMI, proportional amount of body fat). Some studies also demonstrated that NWO people tend to have a low-grade pro-inflammatory status (2, 8–11) and Di Renzo et al. (4) found that normal weight obesity in adult women population was a significant marker of sarcopenia. Some research has also shown that normal weight obesity is present already during childhood (12–15). Wiklund et al. (14) found in their longitudinal study that NWO girls observed for 7 years (from age 11 to 18) had a greater amount of body fat and a stable lean mass/fat mass ratio (LM/FM) index from childhood to adulthood compared to their normal weight and normal BMI peers. Further, NWO girls displayed cardiometabolic risk in childhood, with the risk persisting into early adulthood. Olafsdottir et al. (12) revealed that NWO adolescents had about six kilograms more fat and slightly lower mineral bone density as assessed by DXA. NWO adolescents were also less physically active (assessed by questionnaire) and performed much worse in VO2max than their counterparts with normal weight and normal BMI values. In the area of motor development, when studying the population of preschoolers Musalek et al. (15) found that NWO preschoolers had a significantly poorer degree of fundamental motor skills (FMS), and a more than three times higher risk of severe motor difficulties compared to their NWNO counterparts.

However, apart from previous evidence showing that NWO children and adolescents have a high amount of body fat along with normal BMI and poorer motor performance, there is no information available concerning their skeletal robustness and lean mass development on the extremities. Yet, these parameters are closely related to health development.

The level of general physical activity, exercise, and sport participation—and their sufficiency (character, intensity, and volume)—are the main factors affecting lean mass and bone development (16–20). On the other hand, a low level of lean mass, excessive body fat and poor skeletal development are associated with a decreased level of physical activity (21), a low level of muscular strength and endurance (22) and a higher prevalence of metabolic risk (23, 24) and cardiovascular diseases in child population (25, 26), which continue to adolescence and adult age (14) with serious and worsening health consequences (27). Therefore, it is very important to determine whether skeletal development—robustness and lean mass development on the limbs of NWO children—is significantly weaker compared to their normal weight non-obese peers. And all the more so as previous research in NWO children only looked at more global measures such as the difference in total lean and fat mass or bone mineralization.

The aim of the present study is to investigate the difference in skeletal robustness and lean-fat ratio on the extremities as indicators of health development that are important for: muscular competence, physical fitness and bone health, between NWO aged 9–12, NWNO, and OWOB peers.

## Methods, Subjects

For the purpose of the present study, data from Ministry Research project No. MSM 0021620864 of the Charles University, Faculty of Physical Education and Sport, were used. The data were collected in 2015 from 10 non-specialized elementary schools (i.e., without a specific orientation toward technical studies, the arts, languages, or sport) from Prague, the capital city of the Czech Republic. The data collection was carried out in all schools at the same times, from 9:00 a.m. to 12:00 p.m., over 10 working days in November 2015.

Altogether, 794 middle school children from 9 to 12 years of age were investigated. It is important to say that until today there has been no standard protocol that would provide a definition of normal weight obese children in terms of percentage of body fat and range of BMI. In our study we used two parameters

1. BMI
  - We used BMI percentiles from Czech national BMI reference.
  - We used percentile cut-off points Cole et al. (28) to define normal BMI, overweight and obesity.
2. Skinfolds

Values of the three skinfolds (over triceps, subscapular, suprailliac) were compared with anthropometric references for Czech children (29).

Using the resulting BMI values and measured skinfolds, three categories of children were defined: NWO; OWOB; NWNO children.

The criteria for each defined group of children were as follows:

1. Overweight and obese (OWOB):
  - overweight children: BMI >85th percentile along with average values from three skinfolds >85th percentile of Czech national reference
  - obese children: BMI >95th percentile along with average values from three skinfolds > 95th percentile of Czech national reference
2. Normal weight obese (NWO): BMI 25–60th percentile, along with average values from three skinfolds >85th of Czech national reference; the narrower range of BMI for NWO was selected in order to avoid non-equality of BMI between NWO and NWNO, which is what happened in previous studies. In these studies (4, 12), both NWO and NWNO participants could have BMI in the range of the 25–84th percentile. However, in the end NWO individuals had significantly higher BMI than NWNO peers. Our aim was to select a population of NWO and NWNO that would be indistinguishable based on BMI.
3. Normal weight non-obese individuals (NWNO): BMI in the range of the 25–84th percentile, along with average values from three skinfolds within the 25–84th percentile of Czech national reference (29).

Seven children with abnormal combinations of BMI and skinfold thickness were excluded from the study. These individuals had high BMI (within the range of the 85–90th percentile), along with skinfold values within the range of the 49–58th percentile of the national reference.

A power analysis done in GPower 3.1.3. program showed that when One-way ANOVA (fixed effects, omnibus, one-way); based on Erdfelder, Faul, and Buchner (30), and VanVoorhis and Morgan (31) recommendation with an alpha of 0.05 is used for the three groups (NWO, NWNO, OWOB), a minimum of 159 participants will be required to achieve a size effect of at least (f) 0.25 and power of 80%.

Based on the aforementioned criteria, 72 OWOB children and 69 NWO children were identified from the total studied sample of 787 children. A group of normal weight non-obese children originally included 646 individuals.

In this study our aim was to obtain a research sample which would be as balanced as possible. The main reason was that we wanted to minimize the possible effect of the homogeneity of variance assumption for comparison of defined groups by Analysis of Variance (ANOVA) approach specifically in case of two-way ANOVA. According to (32, 33), both a very unbalanced sample size and heterogeneity variances dramatically affect statistical power and Type I error rates.

To obtain a proportional research sample (*n* = 69) of NWNO children from the total sample of 646 children, a random selection procedure from Randomizer software (www.random.org) was carried out. The research sample finally consisted of 210 middle-school-aged children, from 9 to 11.9 years old (x = 11.3 ± 1.09)

- *n* = 72 Overweight and obese children (OWOB) (boys = 40; girls = 32)
- *n* = 69 Normal weight obese children (NWO) (boys = 26; girls = 43)
- *n* = 69 Normal weight non-obese children (NWNO) (boys = 34, girls = 35)

We realize that the three groups are not fully balanced; however, when using ANOVA, a small violation of balance does not affect the results (31).

Along with the requirement for balanced sample we also analyzed whether the representation of children in each defined category with respect to sex and group did not differ significantly (Table 1).

**Table 1:** Number of participants by age and sex in each of the defined groups.

	**9 years old**		**10 years old**		**11 years old**		**12 years old**	
**Group**	**Boys**	**Girls**	**Boys**	**Girls**	**Boys**	**Girls**	**Boys**	**Girls**
NWNO	*N =* 6	*N =* 8	*N =* 9	*N =* 8	*N =* 9	*N =* 10	*N =* 10	*N =* 9
NWO	*N =* 4	*N =* 9	*N =* 5	*N =* 12	*N =* 8	*N =* 11	*N =* 9	*N =* 11
OWOB	*N =* 6	*N =* 5	*N =* 12	*N =* 7	*N =* 12	*N =* 10	*N =* 10	*N =* 10

*The chi-square test in contingency table rejects significant differences in frequencies of children regarding sex and group: chi-square = 8.14; df = 14; ES = 0.17; p = 0.87*.

*df, degree of freedom; ES, effect size*.

The procedures involved in our study were in accordance with the ethical standards of the responsible Czech national committee on human experimentation and with the Helsinki Declaration of 1975, as revised in 2000. The research was approved by the Ethics Committee of the Faculty of Physical Education and Sport, Charles University, and the parents of all participants signed an informed consent. The data were anonymized.

### Measured Variables

#### Anthropometry

All anthropometric measurements were conducted according to the “Anthropometric Standardization Reference Manual” by Lohman et al. (34) using standardized equipment.

Weight: a medical calibrated scale TPLZ1T46CLNDBI300 was used to assess body weight to the nearest 0.1 kg.

Height: a portable anthropometer P375 (Co. TRYSTOM, spol. s r.o. / 1993-2015 www.trystom.cz) was used. Measurements were taken to the nearest 0.1 cm.

Skinfolds: triceps (tric), subscapular (subsc), suprailiac (suprail), and calf skinfolds were measured with the Harpenden skinfold caliper, with an accuracy of 0.2 mm (35). The latest available data on the thickness of triceps, subscapular, suprailiac, and calf skinfolds for Czech school children were used as references (36).

Skeletal breadth measurements: humeral and femoral epicondyle breadths were measured by the T520 thoracometer (range 0–40 cm) (Co. TRYSTOM, spol. s r.o./1993-2015; http://www.anthropometricinstruments.com/en/modified-thoracometer-t-520/).

Frame indices of skeletal robustness according to Frisancho formula (37) from humerus and femur breadth epicondyles were calculated as follows:

- $\begin{array}{c} \text{Frame index from}\\ \text{upper extremity} \end{array}={\left[\left(\frac{humerus\;epicondyle\;breadth\;in\;mm\;}{body\;height\;in\;cm}\right)\right]}^{*}100$
- $\begin{array}{c} \text{Frame index from}\\ \text{lower extremity} \end{array}={\left[\left(\frac{femur\;epicondyle\;breadth\;in\;mm\;}{body\;height\;in\;cm}\right)\right]}^{*}100$

The body mass index was calculated as follows:

$$\begin{array}{l} \text{BMI =}\frac{weight\;in\;kg\;}{{\left(body\;height\;in\;meters\right)}^{2}} \end{array}$$

*Circumferences: circumferences on the upper* arm and calf were measured by tape measure to the nearest 0.1 cm

Percentage of body fat (%BF): the amount of body fat was calculated according to equations by Slaughter et al. (38). For males with the sum of skinfolds < 35 mm the following equation was used:

$$\begin{array}{l} \%BF=1.21*\left(tric+subsc\right)-0.008*{\left(tric+subsc\right)}^{2}-1.7 \end{array}$$

For females with the sum of skinfolds < 35 mm the following equation was used:

$$\begin{array}{l} \%BF=1.33*\left(tric+subsc\right)-0.013*{\left(tric+subsc\right)}^{2}-2.5 \end{array}$$

For males with the sum of skinfolds higher than 35 mm the following equation was used:

$$\begin{array}{l} \%BF=0.783*\left(tric+subsc\right)+1.6 \end{array}$$

For females with the sum of skinfolds higher than 35 mm the following equation was used:

$$\begin{array}{l} \%BF=0.546*\left(tric+subsc\right)+9.7 \end{array}$$

Slaughter et al. (38)

The muscle area on the upper arm and calf was calculated.

Total upper arm area (TUA)

Upper arm fat area estimate (UFE)

Upper arm muscle area (UMA)

Total calf area (TCA)

Calf fat area estimate (CFE)

Calf muscle area (CMA)

$$\begin{array}{l} TUA=\frac{upper\;arm\;circumference}{\left(4*\pi \right)};\\ UFE=upper\;arm\;circumeference*\frac{triceps\;skinfold}{2}\\ UMA=TUA-UFE\\ TCA=\frac{calf\;circumference}{\left(4*\pi \right)};\\ CFE=calf\;circumference*\frac{calf\;skinfold}{2}\\ CMA=TCA-CFE \end{array}$$

Rolland-Cachera et al. (39)

All anthropometric measurements were taken by one professionally trained researcher from the Faculty of Physical Education and Sport. All raw data were transformed to z-scores. Consequently, all results are presented in z-score normalized values to take account of participants' sex and age.

### Data Analysis

Normality tests included the Shapiro-Wilk test and the Kolmogorov-Smirnov test. The main effects of differences between anthropomorphic characteristics in the sub-groups (OWOB, NWO, and NWNO) were evaluated by the one-way analysis of variance (ANOVA) *p* < 0.05 with probabilities adjusted using sequential Bonferroni corrections. In case significant Bonferroni-corrected main effects were revealed, *post-hoc* comparisons were performed using Fisher's Partial Least Significant Difference so that it could be determined which between-group differences were statistically significant. Along with statistical significance, also the effect size Hays ω^2^ was calculated with the range ω^2^ ≤ 0.059 considered as small effect; ω^2^ 0.059–0.138 as medium effect and ω^2≥^ 0.139 as large effect (40). If effects related to sex were revealed (by two-way ANOVA), then separate ANOVAs were used for boys and girls. When the normality of skinfold values was rejected, the Kruskal Wallis non-parametric ANOVA (*p* < 0.05) was used with *post-hoc* Kruskal-Wallis Multiple-Comparison Z-Value Test (Dunn's Test). Statistical procedures were carried out in the NCSS2007 program (Version 2007; NCSS, Kaysville, UT, USA) (41).

## Results

### Basic Anthropometry: Height, Weight, and BMI

The analysis revealed significant differences in basic anthropometric variables between the defined categories of NWNO, NWO, and OWOB children. Considering height and weight, NWO children were significantly shorter (*p* < 0.01, Hays ω^2^ = 0.10) and lighter (*p* < 0.001, Hays ω^2^ = 0.70) compared to OWOB. No significant differences in the weight and height status were found between NWNO and NWO children. This finding implies that NWNO and NWO children did not differ in their BMI, which is an important assumption for normal weight obesity identification. The average BMI of NWO children corresponded to the 49th percentile of Czech norms for 9–12 years-old children (29). In particular, NWO boys had the average BMI values of the 49th percentile and NWO girls of the 49.25th percentile of the corresponding Czech references. Significantly higher BMI values in both sexes were found in OWOB children (*p* < 0.001, Hays ω^2^ = 0.76) compared to NWO and NWNO peers (Table 2).

**Table 2:** Basic anthropometry characteristic across three assessed groups of NWNO, NWO, and OWOB children.

**Group**	**Z-height Mean/*SD***	**S.E**.	**Z-weight Mean/*SD***	**S.E**.	**Z-BMI Median^†^**	**S.E**.
NWNO	−0.34 ± 1.04	0.12	−0.70 ± 0.47	0.06	−0.69	0.05
NWO	−0.09 ± 0.90	0.11	−0.51 ± 0.45	0.06	−0.58	0.05
OWOB	0.40 ± 0.89^**^	0.10	1.17 ± 0.67^**^	0.07	1.24^**^	0.07

** *p < 0.001 unlike the other two groups*.

† *Results from non parametric Kruskal Wallis ANOVA*.

*SD, standard deviation; S.E., standard error; NOW, normal weight obese; NWNO, normal weight non-obese; OWOB, overweight and obese*.

### Body Fat

**Figure 1:**
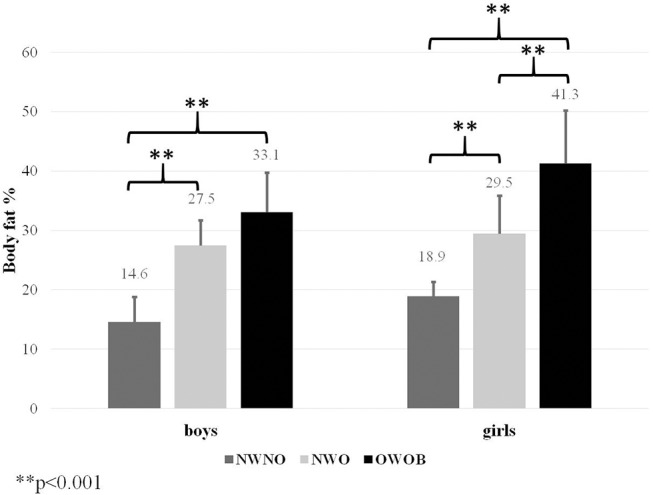
Differences in percentage of body fat estimated by Slaughter equations.

In terms of relative body fat (%), significantly greater values were found in NWO children of both sexes (girls: *p* < 0.001, Hays ω^2^ = 0.65; boys: *p* < 0.001, Hays ω^2^ = 0.62) as compared to their NWNO peers Further, the *post-hoc* Dunn's test for Kruskall Wallis ANOVA also showed that NWO girls (Dunn's test = 3.45) had significantly less amount of body fat compared to OWOB girls. The *post-hoc* analysis between NWO and OWOB boys revealed no significant differences. The greatest value of body fat was revealed in OWOB children (*p* < 0.001) (Figure 1).

### Skeletal Robustness

Further significant differences were also found in skeletal robustness parameters. NWO children had the lowest values of the standard Frame index [estimated on the upper limb according to Frisancho (37)], although the difference was not significant compared to their NWNO peers. OWOB children had by far the highest Frame index calculated on the upper limb Hays ω^2^ = 0.62. Since skeletal robustness does not have to be always symmetrical on the upper and the lower part of the body, we also calculated the Frame index from the lower extremity parameter (breadth of femur epicondyle). This type of index consequently showed that NWO children had significantly poorer robustness on lower limbs as compared to both NWNO and OWOB children (*p* < 0.001, Hays ω^2^ = 0.66). In addition, sex proved to be a major factor affecting robustness of the lower limbs in further analysis [*F*_(2, 205)_ = 8.09, *p* < 0.001]. Among NWO children, the boys had twice poorer robustness of the lower limbs Z-score = −0.85 than NWO girls Z-score = −0.43 as compared to their NWNO and OWOB counterparts (*p* < 0.001; Hays ω^2^ = 0.81) (Table 3).

**Table 3:** Skeletal robustness—Frame indices from humerus and femur breadth epicondyles and the differences between NWO, NWNO, and OWOB children with respect to sex.

**Groups**	**Frame index upper extremity Z-score**		**Frame index lower extremity Z-score**	
	**Mean/*SD***	**S.E**.	**Mean/*SD***	**S.E**.
**BOYS**				
NWNO boys	−0.13 ± 0.99	0.16	0.07 ± 1.0	0.17
NWO boys	−0.60 ± 0.89	0.18	−0.85 ± 0.61^**^	0.13
OWOB boys	0.48 ± 0.87^**^	0.14	0.47 ± 0.89	0.14
**GIRLS**				
NWNO girls	−0.29 ± 1.11	0.19	0.08 ± 0.88	0.15
NWO girls	−0.33 ± 0.88	0.14	−0.43 ± 0.98^*^	0.15
OWOB girls	0.74 ± 0.59^**^	0.10	0.49 ± 0.94	0.17

* *p < 0.01 unlike the other two groups within each sex category*.

** *p < 0.001 unlike the other two groups within each sex category*.

*SD, standard deviation; S.E., standard error; NWO, normal weight obese; NWNO, normal weight non-obese; OWOB, overweight and obese*.

### Lean Mass Development on the Extremities—Muscle Area

The results of muscle areas showed that NWO children had significantly weaker muscle area on the upper arm as well as on the calf compared to NWNO and OWOB counterparts (*p* < 0.001, upper arm Hays ω^2^ = 0.48; calf Hays ω^2^ = 0.59). Further, sex proved to be a major factor. More evidence of the differences in the muscle area on the upper arm and calf was found in boys. OWOB children did not have a significantly different size of the muscle area on the upper arm (Hays ω^2^ < 0.01); however, the size of the muscle area on the calf was significantly poorer compared to the results of NWNO peers (*p* < 0.001, Hays ω^2^ = 0.33, Table 4).

**Table 4:** Upper arm and calf muscle area across three assessed groups of NWNO, NWO, and OWOB children with respect to sex.

**Groups**	**Rolland Cachera upper arm muscle area Z-score**		**Rolland Cachera calf muscle area Z-score**	
	**Mean/*SD***	**S.E**.	**Mean/*SD***	**S.E**.
**BOYS**				
NWNO boys	0.47 ± 0.88	0.11	0.89 ± 0.54	0.10
NWO boys	−1.15 ± 0.71^**^	0.12	−1.34 ± 0.45^**^	0.10
OWOB boys	0.27 ± 0.70	0.14	0.09 ± 0.61	0.09
**GIRLS**				
NWNO girls	0.64 ± 0.51	0.12	0.86 ± 0.55	0.11
NWO girls	−0.95 ± 0.64^**^	0.11	−0.85 ± 0.58^**^	0.12
OWOB girls	0.54 ± 0.82	0.11	0.20 ± 0.94	0.09

** *p < 0.001 unlike the other two groups within each sex category*.

*SD, standard deviation; S.E., standard error; NOW, normal weight obese; NWNO, normal weight non-obese; OWOB, overweight and obese*.

## Discussion

The aim of the present study was to investigate the differences in skeletal robustness and muscle area on the upper arm and calf as an indicator important for: muscular competence, physical fitness, and bone health, which are associated with health development of children, between NWO, NWNO, and OWOB peers aged 9–12 years.

To begin with, we had to find a solution to the problem that was already pointed out by Franco et al. (7), who argued that there was no standard protocol or unified methodology that could be used to identify NWO children. Wiklund et al. (14) used retrospective data from growth charts of relative weight to height gain and value of body fat from DXA. In their study, a NWO individual was defined as an individual with a relative weight between −10% and +20% and body fat ≥30%. On the other hand, Olafsdottir et al. (12) defined NWO adolescents as having BMI in the range of 18.5–24.5 kg/m^2^ along with body fat ≥17.6% for males and ≥31.6% for females referencing the recommendations of Lohman et al. (42). However, these authors did not establish any criteria for relative body fat standards in the NWO population. In adult population, the guidelines for identifying NWO individuals are clearer. NWO adults are those, whose BMI is in the range of 18.5–24.5 kg/m^2^ and who have body fat ≥30%. Some authors (8) even used or recommended gender and age cut-off values (43). Nevertheless, when we looked at previous studies, we found that according to these guidelines NWO participants usually had a significantly higher BMI compared to their normal weight non-obese peers (4, 13). This finding raises the question of whether these people more closely resemble overweight rather than normal weight obese individuals. Therefore, our first major aim in this study was to identify a group of NWO children whose BMI would not differ from BMI of their NWNO counterparts but who will have a significantly greater adipose tissue. Therefore, we defined the range of BMI for NWO as narrower than +- 1SD, specifically from the 25th percentile to the 60th percentile of the national norm. Even though we defined NWNO children as having BMI in the range from the 25th percentile to the 84th percentile of the national norm, in the end the two groups of NWO and NWNO children were indistinguishable from one another by their BMI. In addition, our estimates of body fat that were made using the Slaughter equation were in line with previous studies (2, 4, 6–8, 14), where the amount of body fat of NWO individuals was around 30%.

NWO children of both sexes that we had defined and selected in the manner explained above had the lowest values of skeletal robustness as well as muscle areas on the upper extremity and calf compared to their NWNO and OWOB counterparts. In other words, these children were skeletally more fragile and suffered from weak lean mass development on the extremities. This finding supported the suggestions (44, 45), which pointed out that a strong correlation existed in children between bone area and body weight, lean mass, and fat mass.

Firstly, we shall compare NWO and NWNO children. NWO children displayed significantly weaker lean mass development on the upper arm but did not have significantly poorer skeletal robustness compared to NWNO peers, calculated from humeral epicondyle breadth by Frisancho equation. This could be explained by the function of the upper arm, which is used mainly for manipulation rather than for transportation as is the case of the lower extremities. Warden et al. (46) found that the bone area in humeral diaphysis increases mainly during throwing activities (throwing ball, throwing stone). In addition, several studies reported that during the last few decades children's throwing skills have significantly deteriorated (47) regardless of body status. Therefore, even though NWNO children have better developed lean mass, they probably have comparable throwing skills to their NWO counterparts. On the other hand, NWO children had significantly weaker skeletal robustness calculated as the Frame index from femur breadth epicondyle compared to their NWNO peers. The difference between NWO and NWNO children in weak lean mass development on the calf was even more pronounced. This might suggest that NWO children have little physical activity, in particular transportation activities like walking or running (48). The assumption that physical activity as one of the major drivers of bone area development would support the “mechanostat” hypothesis developed by Frost (49–52) or the results from the Iowa Bone study (17, 18), who proposed that sufficient physical workload and number of muscle contractions are in close relation to bone mass and bone area development. In addition, Slemenda et al. (53) pointed out that increases in calf muscle area are strongly related to bone development and that physical activity is associated with more rapid bone development in prepubertal children. Our results could also provide support for the finding that skeletal robustness in different children populations has been decreasing (21, 54, 55) and the alarming suggestion that the number of NWO children in the population has been rising in the last 20 years. Moreover, when we consider sex as a factor, the skeletal robustness in the lower extremities as well as lean mass development on the calf in NWO boys has declined by an even more significant degree compared to their NWNO counterparts. It could be explained by the fact that boys displayed a greater level and wider range of physical activity, especially vigorous physical activity and also in sport participation (56), compared to girls (57, 58). In other words, we can expect bigger differences in the amount of produced physical activity in boys than in girls. If we accepted the finding of Slemenda et al. (53) about relation between calf muscle area, physical activity and bone development, and also Olafsdottir et al. (12), who revealed that NWO adolescents were less physically active, we could assume that from a long-term perspective the more pronounced weak skeletal robustness in NWO boys could by caused primarily by low physical activity of NWO children. Secondly we compared the results of skeletal robustness and lean mass development of NWO and OWOB children, a typical pattern emerged. OWOB children had significantly greater skeletal robustness estimated both from humeral and femoral epicondyle breadths. A number of previous studies showed that overweight and obese children have greater values of bone development, which is caused mainly by weight (volume of lean mass), height, and biological age (53, 59, 60). Further, OWOB children had significantly higher amount of lean mass on the upper arm and calf in comparison to NWO peers. However, it is interesting to note that OWOB children had significantly smaller amount of lean mass on the calf and only non-significantly higher Frame index calculated from femur epicondyle compared to NWNO counterparts. This would also indicate a low volume of transportation activities like walking or running in these children, which has been well-documented (53, 61, 62). Based on our finding we believe that it would be appropriate to measure under field testing condition also the skeletal robustness from femur epicondyle breadth, which seems to be more sensitive/discriminative compared to the Frame index calculated from humerus epicondyle breadth.

## Conclusion

NWO children (boys and girls) had significantly poorer skeletal robustness on the lower extremities and poorer muscle area on the upper arm and calf as compared to NWNO counterparts. Further, a significantly higher prevalence of poor skeletal robustness as well as poor lean mass development on the lower extremities was found in boys. The highest skeletal robustness—but not muscle area on the lower extremities—was detected in OWOB children. Further research should focus on whether this poor skeletal and lean mass development: (1) is a consequence of insufficient physical activity regimes; (2) affects physical fitness of NWO children and could thus contribute to a higher prevalence of health problems in NWO children. We have highlighted the importance of the development of a simple identification of NWO children that could be used by pediatricians.

## Author Contributions

MM: data collection, introduction, methods, discussion, and conclusion. JP and EG: introdution, methods, and discussion. EB: data analysis and methods. JK: data collection, data analysis, and discussion. JJ: data collection and discussion. ŠV: data analysis and databasing data IT support.

### Conflict of Interest Statement

The authors declare that the research was conducted in the absence of any commercial or financial relationships that could be construed as a potential conflict of interest.

## References

[1] De LA Martinoli R Vaia F Di RL Normal Weight Obese (NWO) Women: an evaluation of a candidate new syndrome. Nutr Metabol Cardiovasc Dis. (2006) 16:513–23. 10.1016/j.numecd.2005.10.010 17126766

[2] De Lorenzo A Del Gobbo V Premrov MG Bigioni M Galvano F Di Renzo L. Normal-weight obese syndrome: early inflammation? Am J Clin Nutr. (2007) 85:40–5. 10.1093/ajcn/85.1.40 17209175

[3] Oliveros E Somers VK Sochor O Goel K Lopez-Jimenez F. The concept of normal weight obesity. Prog Cardiovasc Dis. (2014) 56:426–33. 10.1016/j.pcad.2013.10.003 24438734

[4] Di Renzo L Sarlo F Petramala L Iacopino L Monteleone G Colica C. Association between– 308 G/A TNF-α polymorphism and appendicular skeletal muscle mass index as a marker of sarcopenia in normal weight obese syndrome. Dis. Markers (2013) 35:615–23. 10.1155/2013/983424 24285913 PMC3830785

[5] Ruderman NB Schneider SH Berchtold P The “metabolically-obese,” normal-weight individual. Am J Clin Nutr. (1981) 348:1617–21. 10.1093/ajcn/34.8.1617 7270486

[6] Marques-Vidal P Pécoud A Hayoz D Paccaud F Mooser V Waeber G. Prevalence of normal weight obesity in switzerland: effect of various definitions. Eur J Nutr. (2008) 47:251. 10.1007/s00394-008-0719-6 18604623

[7] Franco LP Silveira AGZ Lima RSDAV Horst MA Cominetti C. APOE genotype associates with food consumption and body composition to predict dyslipidaemia in Brazilian adults with normal-weight obesity syndrome. Clin Nutr. (2017) 37:1722–27. 10.1016/j.clnu.2017.07.002 28720344

[8] Romero-Corral A Somers VK Sierra-Johnson J Korenfeld Y Boarin S Korinek J. Normal weight obesity: a risk factor for cardiometabolic dysregulation and cardiovascular mortality. Eur. Heart J. (2009) 31:737–46. 10.1093/eurheartj/ehp487 19933515 PMC2838679

[9] Madeira FB Silva AA Veloso HF Goldani MZ Kac G Cardoso VC. Normal weight obesity is associated with metabolic syndrome and insulin resistance in young adults from a middle-income country. PLoS ONE (2013) 8:e60673. 10.1371/journal.pone.0060673 23556000 PMC3610876

[10] Renzo L Galvano F Orlandi C Bianchi A Giacomo C Fauci L. Oxidative stress in normal-weight obese syndrome. Obesity (2010) 18:2125–30. 10.1038/oby.2010.50 20339360

[11] Kang S Kyung C Park JS Kim S Lee SP Kim MK. Subclinical vascular inflammation in subjects with normal weight obesity and its association with body fat: an 18 F-FDG-PET/CT study. Cardiovasc Diabetol. (2014) 13:70. 10.1186/1475-2840-13-70 24708764 PMC3994236

[12] Olafsdottir AS Torfadottir JE Arngrimsson SA. Health behavior and metabolic risk factors associated with normal weight obesity in adolescents. PLoS ONE (2016) 11:e0161451. 10.1371/journal.pone.0161451 27560824 PMC4999227

[13] Steffl M Chrudimsky J Tufano JJ. Using relative handgrip strength to identify children at risk of sarcopenic obesity. PLoS ONE (2017) 12:e0177006. 10.1371/journal.pone.0177006 28542196 PMC5441624

[14] Wiklund P Törmäkangas T Shi Y Wu N Vainionpää A Alen M. Normal-weight obesity and cardiometabolic risk: a 7-year longitudinal study in girls from prepuberty to early adulthood. Obesity (2017) 25:1077–82. 10.1002/oby.21838 28429877

[15] Musalek M Kokstejn J Papez P Scheffler C Mumm R Czernitzki AF. Impact of normal weight obesity on fundamental motor skills in pre-school children aged 3 to 6 years. Anthropol Anz. (2017) 74:203–12. 10.1127/anthranz/2017/0752 28765872

[16] Parízková J Body Fat and Physical Fitness: Body Composition and Lipid Metabolism in Different Regimes of Physical Activity. The Hague: Martinus Nijhoff B.V./Medical Division (1977).

[17] Janz KF Burns TL Torner JC Levy SM Paulos R Willing MC. physical activity and bone measures in young children: the iowa bone development study. Pediatrics (2001) 107:1387–93. 10.1542/peds.107.6.1387 11389262

[18] Janz KF Rao S Baumann HJ Schultz JL Measuring children's vertical ground reaction forces with accelerometry during walking, running, and jumping: the iowa bone development study. Pediatr Exerc Sci. (2003) 15:34–43. 10.1123/pes.15.1.34

[19] Parízková J Hills A Childhood Obesity. Prevention and Treatment. 2nd ed Boca Raton, FL; London; New York, NY: CRC Press, Taylor and Francis Group (2010).

[20] Smith JJ Eather N Morgan PJ Plotnikoff RC Faigenbaum AD Lubans DR. The health benefits of muscular fitness for children and adolescents: a systematic review and meta-analysis. Sports Med. (2014) 44:1209–23. 10.1007/s40279-014-0196-4 24788950

[21] Rietsch K Eccard JA Scheffler C. Decreased external skeletal robustness due to reduced physical activity? Am J Hum Biol. (2013) 25:404–10. 10.1002/ajhb.22389 23606229

[22] Ortega FB Ruiz JR Castillo MJ Sjöström M. Physical fitness in childhood and adolescence: a powerful marker of health. Int J Obes. (2008) 32:1. 10.1038/sj.ijo.0803774 18043605

[23] Benson AC Torode ME Fiatarone Singh MA. Muscular strength and cardiorespiratory fitness is associated with higher insulin sensitivity in children and adolescents. Int J Pediatr Obes. (2006) 1:222–31. 10.1080/17477160600962864 17907329

[24] Steene-Johannessen J Anderssen SA Kolle E Andersen LB. Low muscle fitness is associated with metabolic risk in youth. Med Sci Sports Exer. (2009) 41:1361–7. 10.1249/MSS.0b013e31819aaae5 19516166

[25] Freedman DS Mei Z Srinivasan SR Berenson GS Dietz WH. Cardiovascular risk factors and excess adiposity among overweight children and adolescents: the Bogalusa Heart Study. J Pediatr. (2007) 150:12–7. 10.1016/j.jpeds.2006.08.042 17188605

[26] Magnussen CG Schmidt MD Dwyer T Venn A. Muscular fitness and clustered cardiovascular disease risk in australian youth. Eur J Appl Physiol. (2012) 112:3167–71. 10.1007/s00421-011-2286-4 22183088

[27] Daniels SR. The consequences of childhood overweight and obesity. Future Child. (2006) 16:47–67. 10.1353/foc.2006.0004 16532658

[28] Cole TJ Flegal KM Nicholls D Jackson AA. Body mass index cut offs to define thinness in children and adolescents: international survey. Br Med J. (2007) 335:194–201. 10.1136/bmj.39238.399444.55 17591624 PMC1934447

[29] Vignerová J Riedlová J Bláha P Kobzová J Krejčovský L Brabec M. 6. Celostátní antropologický výzkum dětí a mládeŽe 2001 Ceská republika. In: Souhrnné výsledky. 6th Nation-wide Anthropological Survey of Children and Adolescents 2001 Czech Republic. Summary Results (2006). 12461491

[30] Erdfelder E Faul F Buchner A GPOWER: A General Power Analysis Program. Behav Res Methods Instr Comput. (1996) 28:1–11. 10.3758/BF03203630

[31] VanVoorhis CW Morgan BL Understanding power and rules of thumb for determining sample sizes. Tutor Quant Methods Psychol. (2007) 3:43–50. 10.20982/tqmp.03.2.p043

[32] Rusticus SA Lovato CY Impact of sample size and variability on the power and type I error rates of equivalence tests: a simulation study. Prac Assess Res Eval. (2014) 19:2.

[33] Milliken GA Johnson DE Analysis of Messy Data, Volume I: Designed experiments. Belmont, CA: Wadsworth. Inc. (1984).

[34] Lohman TG Roche AF Martorell R Anthropometric Standardization Reference Manual. Champaign, IL: Human Kinetics Books (1988).

[35] Carter JL Heath BH Somatotyping: Development and Applications. Vol. 5. Cambridge: Cambridge University Press (1990).

[36] Vignerová J Bláha P Sledování rustu českých dětí a dospívajících. Norma, vyhublost, obezita 1. vydání. Praha, Státní zdravotní ústav, Translation: Investigation of Growth in Czech Children and Adolescence. Norm, Emaciation, Obesity. 1st ed. Praha: Státní zdravotní ústav (2001).

[37] Frisancho AR Anthropometric Standards for the Assessment of Growth and Nutritional Status. Michigan: University of Michigan Press (1990).

[38] Slaughter MH Lohman TG Boileau R Horswill CA Stillman RJ Van Loan MD. Skinfold equations for estimation of body fatness in children and youth. Hum. Biol. (1988) 60:709–23. 3224965

[39] Rolland-Cachera MF Brambilla P Manzoni P Akrout M Sironi S Del Maschio A. Body composition assessed on the basis of arm circumference and triceps skinfold thickness: a new index validated in children by magnetic resonance imaging. Am J Clin Nutr. (1997) 65:1709–13. 10.1093/ajcn/65.6.1709 9174464

[40] Kirk RE Practical significance: a concept whose time has come. Educ. Psychol. Meas. (1996) 56:746–59.

[41] Hintze J NCSS 2007. NCSS, LLC. Kaysville,UT. USA. Available online at: www.ncss.com (2007).

[42] Lohman TG Houtkooper L Going SB Body fat measurement goes high-tech: not all are created equal. ACSM's Health Fit J. (1997) 1:30–5.

[43] Gallagher D Heymsfield SB Heo M Jebb SA Murgatroyd PR Sakamoto Y. Healthy percentage body fat ranges: an approach for developing guidelines based on body mass index–. Am J Clin Nutr. (2000) 72:694–701. 10.1093/ajcn/72.3.694 10966886

[44] Ilich JZ Skugor M Hangartner T Baosh A Matkovic V. Relation of nutrition, body composition and physical activity to skeletal development: a cross-sectional study in preadolescent females. J Am Coll Nutr. (1998) 17:136–47. 10.1080/07315724.1998.10718739 9550457

[45] Sabatier JP Guaydier-Souquieres G Benmalek A Marcelli C. Evolution of lumbar bone mineral content during adolescence and adulthood: a longitudinal study in 395 healthy females 10–24 years of age and 206 premenopausal women. Osteopor Int. (1999) 9:476–82. 10.1007/s001980050173 10624453

[46] Warden SJ Roosa SMM Kersh ME Hurd AL Fleisig GS Pandy MG. Physical activity when young provides lifelong benefits to cortical bone size and strength in men. Proc Natl Acad Sci USA. (2014) 111:5337–42. 10.1073/pnas.1321605111 24706816 PMC3986122

[47] Sedlak P Parízková J Daniš R Dvoráková H Vignerová J. Secular changes of adiposity and motor development in Czech preschool children: Lifestyle changes in fifty-five year retrospective study. BioMed Res Int. (2015) 2015:823841. 10.1155/2015/823841 26380296 PMC4561935

[48] Sedlak P Parízková J Procházková L Cvrčková L Dvoráková H. Secular changes of adiposity in czech children aged from 3 to 6 years: latent obesity in preschool age. Biomed Res. Int. (2017) 2017:2478461. 10.1155/2017/2478461 29270426 PMC5706086

[49] Frost HM. Bone “mass” and the “mechanostat”: a proposal. Anatom Record (1987) 219:1–9. 3688455 10.1002/ar.1092190104

[50] Frost HM The Laws of Bone Structure. Springfield, IL: Charles C. Thomas (1964).

[51] Frost HM Bone Modeling and Skeletal Modeling Errors. Springfield: Charles C. Thomas (1973).

[52] Frost HM. Why do marathon runners have less bone than weight lifters? A vital-biomechanical view and explanation. Bone (1997) 20:183–9. 9071467 10.1016/s8756-3282(96)00311-0

[53] Slemenda CW Reister TK Hui SL Miller JZ Christian JC Johnston CC Jr. Influences on skeletal mineralization in children and adolescents: evidence for varying effects of sexual maturation and physical activity. J Pediatr. (1994) 125:201–7. 8040762 10.1016/s0022-3476(94)70193-8

[54] Rietsch K Godina E Scheffler C Decreased external skeletal robustness in schoolchildren–a global trend? Ten year comparison of Russian and German data. PLoS ONE (2013) 8:e68195 10.1371/journal.pone.0068195 23935857 PMC3720668

[55] Scheffler C. The change of skeletal robustness of 6-12 years old children in Brandenburg (Germany)-comparison of body composition 1999-2009. Anthropol Anzeig. (2011) 68:153–65. 10.1127/0003-5548/2011/0095 21452680

[56] Trost SG Pate RR Sallis JF Freedson PS Taylor WC Dowda M. Age and gender differences in objectively measured physical activity in youth. Med Sci Sport Exerc. (2002) 34:350–5. 10.1097/00005768-200202000-00025 11828247

[57] Vilhjalmsson R Kristjansdottir G. Gender differences in physical activity in older children and adolescents: the central role of organized sport. Soc Sci Med. (2003) 56:363–74. 10.1016/S0277-9536(02)00042-4 12473321

[58] Marques A Ekelund U Sardinha LB. Associations between organized sports participation and objectively measured physical activity, sedentary time and weight status in youth. J Sci Med Sport (2016) 19:154–7. 10.1016/j.jsams.2015.02.007 25766508 PMC6235112

[59] Goulding A Taylor RW Jones IE McAuley KA Manning PJ Williams SM. Overweight and obese children have low bone mass and area for their weight. Int J Obes. (2000) 24:627. 10.1038/sj.ijo.0801207 10849586

[60] Petit MA Beck TJ Shults J Zemel BS Foster BJ Leonard MB. Proximal femur bone geometry is appropriately adapted to lean mass in overweight children and adolescents. Bone (2005) 36:568–76. 10.1016/j.bone.2004.12.003 15777684

[61] Marten N Olds T Physical activity: patterns of active transport in 11–12 year old Australian children. Aust N Z J Public Health (2004) 28:167–72. 10.1111/j.1467-842X.2004.tb00931.x 15233357

[62] Jiménez-Pavón D Kelly J Reilly JJ. Associations between objectively measured habitual physical activity and adiposity in children and adolescents: systematic review. Int J Pediatr Obes. (2010) 5:3–18. 10.3109/17477160903067601 19562608

